# The Application of GHRH Antagonist as a Treatment for Resistant APL

**DOI:** 10.3390/cancers15123104

**Published:** 2023-06-08

**Authors:** Ravinder S. Chale, Stephanie M. Almeida, Mario Rodriguez, Ivan Jozic, Simonetta I. Gaumond, Andrew V. Schally, Joaquin J. Jimenez

**Affiliations:** 1 Dr. Phillip Frost Department of Dermatology and Cutaneous Surgery, Miller School of Medicine, University of Miami, 1600 NW 10th Ave RMSB R250, Miami, FL 33136, USAsxg1204@miami.edu (S.I.G.);; 2 Division of Endocrinology, Department of Medicine, Miller School of Medicine, University of Miami, Miami, FL 33136, USA; 3 Veterans Affairs Medical Center, Miami, FL 33125, USA; 4 Sylvester Comprehensive Cancer Center, Department of Medicine, Miller School of Medicine, University of Miami, Miami, FL 33136, USA; 5 Division of Hematology/Oncology, Department of Medicine, Miller School of Medicine, University of Miami, Miami, FL 33136, USA; 6 Department of Pathology, Miller School of Medicine, University of Miami, Miami, FL 33136, USA; 7 Department of Biochemistry and Molecular Biology, Miller School of Medicine, University of Miami, Miami, FL 33136, USA

**Keywords:** APL, AML, resistance, growth hormone releasing hormone, MIA-602

## Abstract

**Simple Summary:**

Previously, it has been shown that the use of growth hormone–releasing hormone (GHRH) antagonistic peptide analogs significantly suppresses the proliferation of various human cancer cell lines. However, the potential of the GHRH antagonist MIA-602 in addressing the resistance of acute promyelocytic leukemia (APL), as well as its ability to produce synergistic effects in acute myeloid leukemia (AML), have not yet been studied. Our findings indicate that the APL double-resistant cell line (NB4-RAA) and the K-562 AML cell line possess the GHRH receptor (GHRH-R) and are thus susceptible to treatment. We further described neural cell adhesion molecule 1 (NCAM1 classified as CD56) and its association with resistance to standard APL treatment in our in vitro model.

**Abstract:**

GHRH is a hypothalamic peptide shown to stimulate the proliferation of malignant cells in humans. We have previously shown that the use of GHRH antagonist MIA-602 successfully suppressed the growth of many human cancer cell lines, spanning more than 20 types of cancers. In this study, we demonstrate the presence of GHRH-R in the NB4, NB4-RAA, and K-562 model cell lines. Furthermore, we demonstrate the inhibited proliferation of all three cell lines in vitro after incubation with MIA-602. The treatment of xenografts of human APL cell lines with MIA-602 led to a significant reduction in tumor growth. Additionally, combination therapy with both doxorubicin (DOX) and MIA-602 showed a marked synergistic effect in reducing the proliferation of the K-562 AML cell line. These findings suggest that MIA-602 could be utilized to address resistance to all-*trans* retinoic acid (ATRA) and arsenic trioxide (ATO) therapies, as well as in augmenting anthracycline-based regimens.

## 1. Introduction 

APL is a rare form of AML that accounts for approximately 10–15% of all AML cases [1]. This subtype of leukemia is characterized by hyper-acute onset, hemorrhagic occurrences, and an increase in promyelocyte proliferation [1]. 

In clinical practice, the combined use of ATRA and ATO is considered the standard treatment for APL [2,3]. ATO has been used to overcome ATRA resistance in patients with APL [4]. However, resistance to ATO has now emerged as an important clinical problem with limited treatment options available [2,5,6,7]. An inability to respond effectively to both ATRA and ATO therapies has been linked with an increased risk of mortality [3,8]. Additionally, disease recurrence has been observed in approximately 10–20% of APL patients undergoing the standard treatment [7,9,10,11]. 

The use of differentiation-inducing agents, such as ATRA in combination with ATO, in APL can lead to a life-threatening condition such as differentiation syndrome, which is observed in approximately 20–25% of patients [3,12]. Although ATO remains the most effective treating agent for ATRA-resistant APL, no single-agent therapy exists to treat ATO-resistant APL, which is associated with a high mortality rate [13,14]. Thus, identifying the underlying mechanisms of ATRA and ATO resistance, particularly in high-risk patients, is of critical priority and remains a significant clinical problem in APL therapy [15,16].

Neural cell adhesion molecule 1 (NCAM1 classified as CD56) is typically considered as a primary phenotypic indicator of natural killer cells. However, it can also be found on various immune cells such as alpha beta T cells, gamma delta T cells, dendritic cells, and monocytes. In individuals with malignant diseases and other autoimmune afflictions, there have been reports of deficiencies in both the numerical and functional aspects of the CD56+ immune cell population [17].

Research findings suggest that the presence of CD56 is linked to drug resistance in AML patients, with up to 20% expressing this marker [8,18]. Moreover, CD56 has been demonstrated to play an essential role in the regulation of cell survival and stress resistance [8]. 

CD56 expression is associated with the constitutive activation of the MAPK-signaling pathway, the regulation of apoptosis, and with glycolysis [8]. When activated, CD56 is known to induce a range of signaling cascades such as FYN–focal adhesion kinase (FAK), MAPK, and phosphatidylinositol 3-kinase (PI3K) pathways [8]. The aberrant expression of CD56 is associated with decreased complete remission rates, higher rates of relapse, and poor overall survival in approximately 15–20% of AML patients [8]. The significance of CD56 as a prognostic marker is particularly notable in AML or APL patients harboring t (8;21) [8,19]. Furthermore, the presence of CD56 is also associated with a higher probability of extramedullary disease and hyperleukocytic syndromes [8,19].

The GHRH peptide, produced in the hypothalamus, stimulates the anterior pituitary to produce and release human growth hormone [20]. Many human tumors have been found to produce GHRH, which acts as an autocrine/paracrine growth factor to promote proliferation. Previously, we have demonstrated that GHRH-R is expressed in human AML cell lines (K-562, THP-1, and KG-1a) [21]. The GHRH peptide has also been identified in samples of surgical tissues from various types of malignancies such as breast, endometrial, ovarian, colorectal, gastric, pancreatic, lung cancers, lymphomas, and other related human cancer cell lines [22,23]. The expression of GHRH-R in a variety of tumors supports a novel treatment strategy based on GHRH antagonists [21,24,25]. We have previously produced nearly 2000 synthetic antagonistic analogues of GHRH [24,26], which have been shown to inhibit, with few adverse effects, the development of 60 human cancer cell lines xenografted into nude mice [26].

This study aims to investigate the impact of MIA-602, a GHRH antagonist, on human promyelocytes that have acquired resistance to both ATRA and ATO. Additionally, this study aims to explore the role of MIA-602 in treating the AML cell line K-562.

## 2. Materials and Methods

### 2.1. Peptides and Reagents 

As previously described (Zarandi et al. (1994, 2017)), MIA-602 was produced using the synthesized solid-phase method and purified using reverse-phase high-performance liquid chromatography (HPLC) [24]. The chemical composition of MIA-602 is [PhAc-Ada)^0^-Tyr^1^, D-Arg^2^, Fpa5^6^, Ala^8^, Har^9^, Tyr (Me)^10^, His^11^, Orn^12^, Abu^15^, His^20^, Orn^21^, Nle^27^, D-Arg^28^, Har^29^] Hgh-RH (1-29) NH_2_. Non-coded amino acids and acyl groups are abbreviated as follows: Abu, alpha-aminobutyric acid; Ada, 12-aminodode-canoyl; Fpa5, pentafluoro-phenylalanine; Har, homoarginine; Nle, norleucine; Orn, ornithine; PhAc, phenylacetyl; and Tyr(Me), *O*-methyl-tyrosine. In vitro, the peptide was dissolved in dimethyl sulfoxide (DMSO) and diluted with incubation media, with a final concentration of DMSO never exceeding 0.1%. In vivo studies used MIA-602 dissolved in DMSO, which was then diluted with sterile aqueous phosphate-buffered saline (PBS) Ph 7.4 (1X) solution, which served as the vehicle control. 

### 2.2. Cell Culture

K-562 (CCL-243, ATCC) are a highly undifferentiated type of lymphoblast cells obtained from the bone marrow of a patient with chronic myelogenous leukemia [27]. The K-562 cell line was obtained from the American Type Culture Collection (ATCC, Manassas, VA, USA). NB4 is an APL cell line derived from the bone marrow of a patient with APL [28,29,30]. The NB4-RAA (ATRA and ATO resistant NB4) cell line was developed in Dr. Jimenez’s Lab from the parent NB4 cell line by culturing NB4 in the presence of increasing concentrations of ATRA and ATO. This process required multiple subcultures over a period of twelve months. NB4 and NB4-RAA were cultured in suspension in RPMI-1640 and 10% fetal calf serum. K-562 were maintained in Iscove’s modified Dulbecco’s medium (IMDM). All media were supplemented with 2 mmol/L L-Glutamine, 25 mmol/L HEPES, 10% FBS, and 50 Lg/mL Gentamicin. Cells were cultured in an incubator at 5% CO_2_ with 100% humidity at 37 °C. 

### 2.3. Cell Proliferation

For K-562 cells, IMDM supplemented with 1% Fetal Calf Serum, 2 mmol/L L-Glutamine, 25 mmol/L HEPES, and 50 µg/mL Gentamicin was used. For NB4 and NB4-RAA cells, RPMI 1640 supplemented with 1% Fetal Calf Serum, 2 mmol/L L-Glutamine, 25 mmol/L HEPES, and 50 µg/mL Gentamicin was used. Microplates of 12 wells were used, and each well was seeded with 2.5 × 10^5^ cells/Ml. MIA-602 was then added at a concentration of 0.05, 0.5, and 5 µmol/L, and cell counts were taken at 24 and 48 h. Quadruplicate measurements were taken for each concentration and the control group, and the MOXI mini–Automated Cell Counter (Orflo Technologies, Ketchum, ID, USA) was utilized for cell counting. 

### 2.4. Apoptosis Assay

The rate of apoptosis was evaluated using the terminal deoxynucleotidyl transferase-mediated Dutp nick end labeling (TUNEL) method (DeadEnd™ Fluorometric TUNEL System, Protocol, Promega, Madison, WI, USA). The apoptotic and live cells were then quantified using a fluorescent microscope according to the manufacturer’s instructions.

### 2.5. In Vivo Experiments in Mice

Female athymic nude mice (Hsd: AthymicNude-Foxn1^nu^) at eight weeks of age were soured from Harlan Laboratories (Indianapolis, IN, USA) and were maintained in sterile cages under temperature-controlled conditions. The mice were provided with autoclaved chow and water ad libitum. Leukemia cells NB4 and NB4-RAA were introduced through subcutaneous (s.c.) injection of 1 × 10^7^ cells. When the tumors grew to an estimated size (approx. 40 mm^3^), the mice were randomized into two groups consisting of 6 animals each. Depending on their group assignment, they were given s.c. injections of either PBS (100 µL) solution containing 0.1% DMSO (Control) or MIA-602 at a dose of 10 µg, twice a day in their left flank. Tumor volume (length × width × height × 0.5236) and bodyweight were measured starting on day one of PBS or MIA-602 injection and every 7 days. All animal procedures were carried out in accordance with the National Institutes of Health Guide for the Care and Use of Laboratory Animals and were approved by the Animal Care and Use Committee of the University of Miami. Results are expressed as means ± SEM, with n = 6 mice per group.

### 2.6. Western Blotting

To prepare whole cell lysates, NB4, NB4-RAA, and K-662 cells were washed twice with ice-cold PBS and lysed in ice-cold lysis buffer (20 Mm Tris-HCl Ph 7.5, 150 Mm NaCl 1% Triton X-100) (PMID: 34145387). After clarification by centrifugation, the lysates underwent protein concentration, determined via the BCA Protein Assay Reagent Kit (Thermo Scientific, Waltham, MA, USA). The proteins were then resolved using 4–20% Criterion TGX pre-cast gels (Bio-Rad, Hercules, CA, USA) and subsequently transferred onto polyvinylidene difluoride membranes (Thermo Scientific). Next, they were placed in blocking buffer for 1 h, (TBS, 0.1% Tween20, 5% BSA), followed by overnight incubation with specified antibodies. 

### 2.7. Statistical Analysis

The statistical analysis was conducted using GraphPad Prism 6 software (GraphPad Software, San Diego, CA, USA). The results were presented as means +/− standard error of the mean (SEM), with significance accepted at *p* < 0.05. One-way analysis of variance (ANOVA) was performed followed by Dunnett’s post hoc test.

## 3. Results

**Figure 1:**
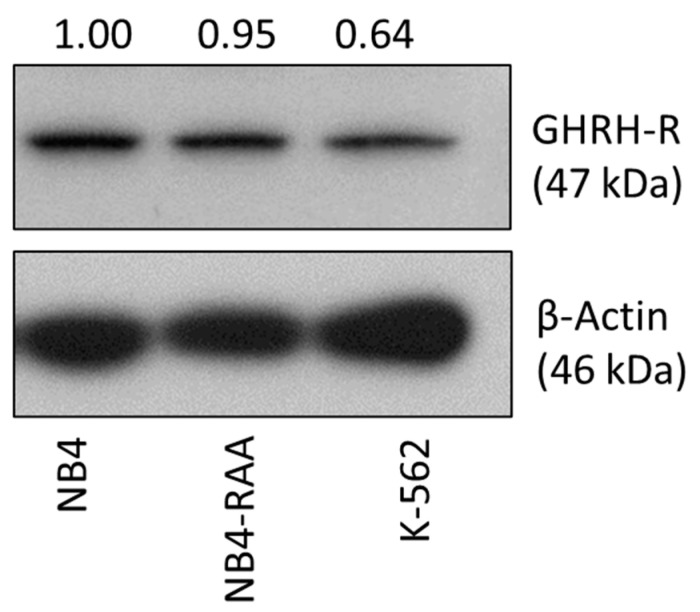
Western Blot analysis showing expression of GHRH-R in NB4, K-562, and NB4-RAA cell lines. GHRH-R was present at 47 kDa in the three cell lines with relative abundance quantified by densitometry normalized to β-Actin. NB4: parent cell line; NB4-RAA: NB4 ATRA+ATO double-resistant cell line; K-562: AML cell line. Original western blots are presented in Supplementary Material File S1.

To evaluate the susceptibility of MIA-602 targeted therapy, we initially examined the expression of GHRH-R in NB4 and NB4-RAA cells. The K-562 AML cell line was used due to its previously established suitability for AML modeling and our prior demonstration of GHRH-R expression [1,21]. A Western Blot was conducted using an antibody specific to a C-terminal region of the pituitary type GHRH-R, which is expressed by both NB4 and NB4-RAA cells (Figure 1). Based on the positive expression of GHRH-R in the NB4, NB4-RAA, and K-562 cell lines used, we hypothesized that MIA-602 could be effective in overcoming APL resistance and serving as an adjuvant therapy in AML. 

**Figure 2:**
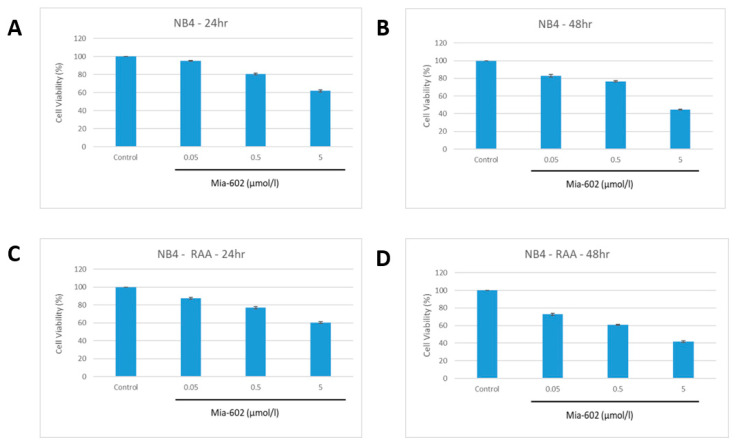
Decreased proliferation of NB4 parent (**A**,**B**) and NB4-RAA double resistant (**C**,**D**) cell lines at 24 and 48 h with increasing concentrations of MIA-602. No observed difference in cell viability was found between NB4 parent and NB4-RAA resistant cell lines when treated with MIA-602 GHRH-R antagonist (*p* < 0.05). NB4: parent cell line, NB4-RAA: NB4 ATRA+ATRO double resistant.

Following this, NB4 and NB4-RAA cells were grown in the presence of MIA-602 concentrations ranging from 0.05 to 5 µmol/L for 24 and 48 h. As shown in Figure 2, the treatment with MIA-602 caused a significant dose- and time-dependent decrease in cell proliferation in the two GHRH-R-positive cell lines. Both cell lines showed comparable reductions in their ability to survive when exposed to MIA-602 concentrations greater than 0.05 μmol/L (*p* < 0.05) after 24 and 48 h. 

At concentrations exceeding 0.5 μmol/L of MIA-602, a noticeable decrease in cell survival was observed after 48 h. When exposed to 5 μmol/L of MIA-602, no viable cells were detected in either of the cell lines after 48 h. However, there was no observed difference in cell viability between NB4 and MIA-602-resistant NB4-RAA when subjected to equal concentrations of MIA-602.

The percentage of apoptotic cells was measured with TUNEL after 48 h of treatment with 5 µmol/L MIA-602. For untreated-NB4 cells, 2% apoptosis was observed (SEM = 1%), whereas for MIA-602-treated cells, 71% apoptosis was observed (SEM = 2.5%). Similarly, for untreated-NB4-RAA cells, 2% apoptosis was observed (SEM = 1%), whereas for MIA-602-treated cells, 78% apoptosis was observed (SEM = 3%). In K562 cells, the control condition showed 1% apoptosis (SEM = 0.5%), while MIA-602-treated cells showed between 75% and 80% apoptosis (SEM = 3.5%). 

**Figure 3:**
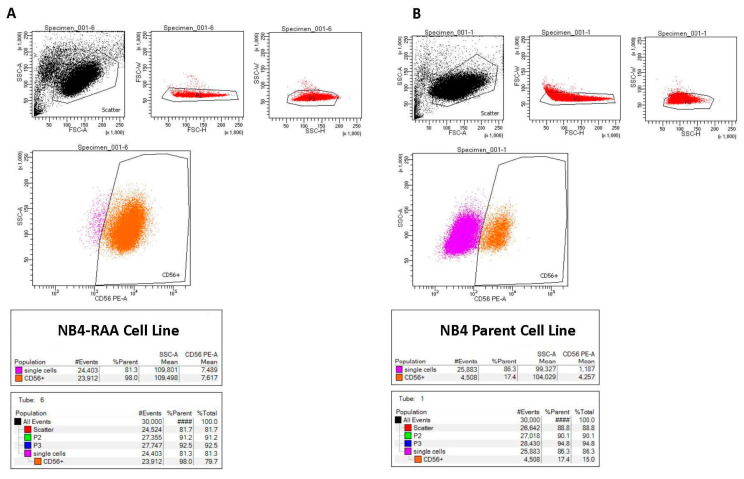
Flow cytometry results showing significantly increased expression of CD56 in the NB4-RAA cell line (**A**) compared to the NB4 parent cell line (**B**), (98% vs. 17.4%), (>5.8-fold; *p* < 0.01).

Flow cytometry analysis revealed a notable rise in CD56 expression (>5.8-fold; *p* < 0.05) in NB4-RAA cells compared to the parent cell line (Figure 3). Although the NB4-RAA cell line exhibited a significant upregulation of the CD56 neural adhesion factor, no difference in susceptibility to MIA-602 or cell viability was observed compared to the treatment parent cell line. 

**Figure 4:**
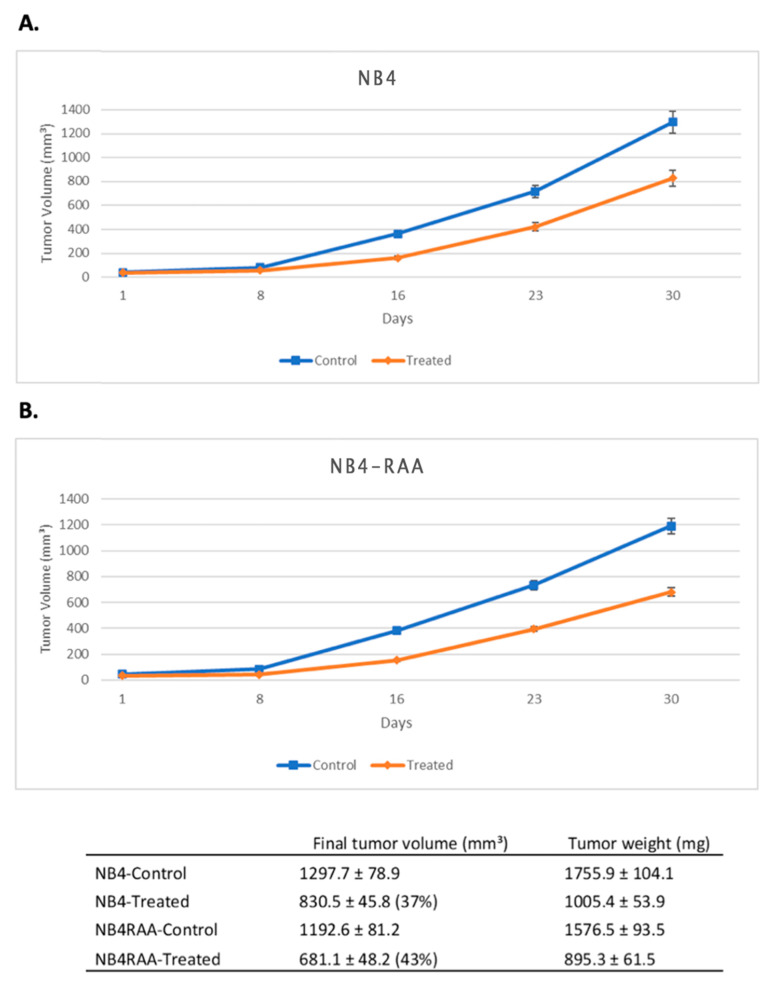
GHRH-R is expressed in xenografted tumors of human APL cell lines NB4 and NB4-RAA, and these tumors respond to treatment with GHRH antagonist MIA-602. Tumors were harvested from untreated mice 4 weeks after the injection of leukemia cells. The scalebar (**A**,**B**) is 100 µm. The effect of MIA-602 treatment on the growth of NB4 (**A**) and of NB4-RAA (**B**) tumors is depicted above. The orange line represents mice that received MIA-602 treatment (10 µg, twice a day for 30 days), while the blue line represents untreated control animals. Results are presented as means +/− SEM, with n = 6 mice per group.

To create an in vivo model, the parent NB4 and NB4-RAA cell lines were xenografted by s.c. injection into athymic nude mice. MIA-602 was given s.c. at a dose of 10 µg twice a day for 30 days. Treatment with MIA-602 at this dose significantly (*p* < 0.05) reduced the final volume of NB4 treated and NB4-RAA treated tumors by 37% and 43%, respectively, and prolonged the tumor doubling time (Figure 4). No significant differences in bodyweights or weights of various organs (liver, spleen, kidneys) were observed between treated animals and non-tumor-bearing animals. A macroscopic examination of organs did not reveal any difference post-treatment with MIA-602.

**Figure 5:**
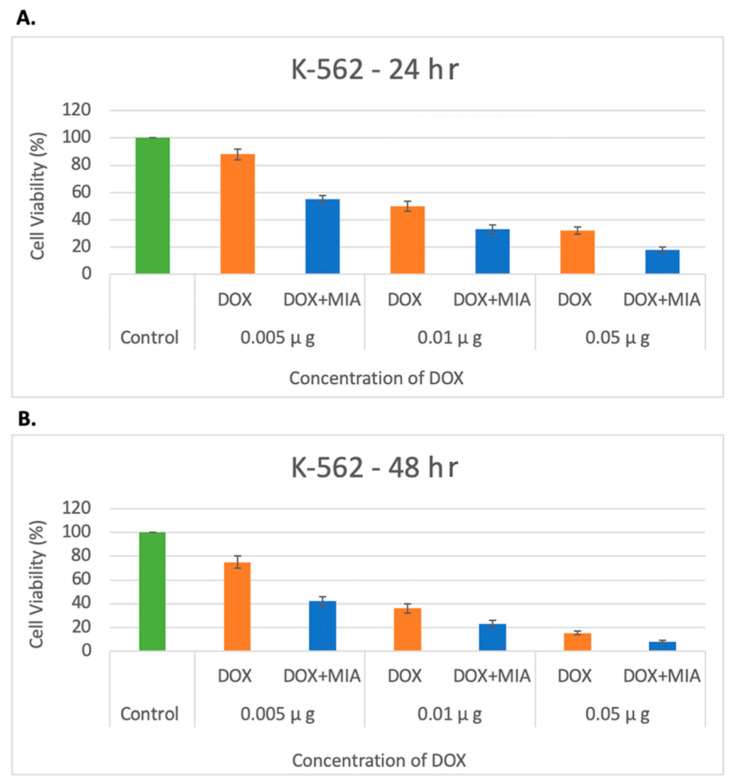
Decreased proliferation of K-562 cells under treatment with DOX and DOX + MIA-602 at 24 h (**A**) and 40 h (**B**) by concentrations of 0.005, 0.01 and 0.05 µg/Μl of DOX. Cell viability decreased as concentration of DOX increased with an additive effect after the addition of antagonist MIA-602 in all concentrations of DOX. A total of 5 µmol/L of MIA-602 was used in all combinations. A dose-dependent reduction in viability was seen at all concentrations of DOX. A significantly increased reduction in viability was seen when combination therapy was used at all concentrations at 24 and 48 h. The most effective dose was seen with 0.05 µg/μL DOX and 5 µmol/L of MIA-602. DOX: orange; DOX + MIA-602: blue; Control: green.

Next, the potential for synergistic effects of MIA-602 in combination with DOX in treating AML was assessed by utilizing an in vitro AML model with the K-562 cell line. K-562 cells were seeded at a density of 2.5 × 10^4^ cells/Ml and treated with DOX at concentrations of 0.005, 0.01, and 0.05 μg/Μl. The combination therapy concentrations were 0.005, 0.01, and 0.05 μg/Μl for DOX and 5 μmol/L for MIA-602. The viability was observed and recorded at 24 and 48 h. An incubation with DOX resulted in a dose-dependent decrease in viability. A co-incubation with MIA-602 and DOX led to a significantly greater reduction in viability (*p* < 0.05). Moreover, the co-administration of DOX and MIA-602 demonstrated a synergistic effect, when compared to DOX alone at all concentrations and time points. The combination of 0.05 µg/Μl of DOX and 5 μmol/L of MIA-602 was found to be the most effective dose in reducing proliferation (Figure 5).

## 4. Discussion

Our results indicate that the use of GHRH antagonists, such as MIA-602, could be a viable strategy for treating APL and AML that are resistant to standard therapies.

Previous research has shown that MIA-602 and other GHRH antagonists can inhibit the growth, tumorigenicity, and metastases of various human experimental cancers by targeting GHRH-R [21,25,26,31]. Our results demonstrate that GHRH-R is expressed in both the NB4 APL model cell line and K-562 AML model. Moreover, the acquired resistance to ATRA and ATO did not affect susceptibility to the MIA-602 GHRH antagonist, nor did it disrupt the expression of the GHRH-R in our in vitro model. 

Given the clinical significance of increased expression of CD56, and its role in drug resistance, poor overall survival rates, and higher relapse rates, we proceeded to check for the expression of this marker in our in vitro model [8]. The expression of CD-56 was significantly increased in resistant NB4 cells when compared to its parent cell line. An acquired resistance to ATRA/ATO may, in part, be mediated by the signaling cascades associated with the increased expression of CD-56 such as MAPK, PI3K, or the regulation of apoptosis. Whether MIA-602 downregulates these pro-apoptotic pathways in resistant APL has yet to be investigated and may be the focus of future research. Our findings demonstrate that the use of MIA-602 in the inhibition of proliferation, in vitro as well as in our preclinical mouse model of resistant APL, has important therapeutic implications. Further studies are necessary to examine the clinical utility of targeting GHRH-R in the APL- and AML-resistant patient populations. 

We have previously shown that MIA-602 can impede the growth of human myeloid leukemic cell lines by increasing the expression of proapoptotic genes such as CAS9 and tumor necrosis factor (TNF), while simultaneously inactivating Akt [21,22,24]. The antiproliferative effect of MIA-602 is partly mediated through the upregulation of pro-apoptotic pathways. Additionally, our previous studies have demonstrated the inhibition of GHRH from binding to its respective receptors in tumors as well as the downregulation of the GHRH-R [24]. In vitro models of gastric cancer and xenografted tumors have also shown that MIA-602 can downregulate the PAK-1-mediated STAT3/NF-κB inflammatory pathway [31]. This pathway may mediate the pro-apoptotic effects observed in this study. Notably, MIA-602 elicits a distinct apoptotic pathway from that of ATRA and ATO. We have shown that, despite resistance to front line ATRA/ATO therapy, NB4-RAA cells remain susceptible to MIA-602. Therefore, MIA-602 may serve as an important therapeutic option for the high-risk subset of patients with relapsed APL. The use of MIA-602 can be especially useful in the treatment of resistant APL, as those who become resistant to ATO have an increased risk of mortality [1,5,6,32].

When co-administered with DOX, the GHRH antagonist MIA-602 showed a synergistic effect in decreasing the proliferation of the K-562 AML cell line. After a 48 h period, the combination of MIA-602 and DOX exhibited a significant reduction in proliferation, in comparison to DOX treatment alone. The use of MIA-602 with chemotherapy-based regimens may be beneficial in preventing the development of resistance in AML, while simultaneously minimizing adverse effects, as MIA-602 is a non-cytotoxic agent. Additional research, focused on understanding the specific molecular pathways responsible for the synergistic effect of MIA-602, could yield valuable information for enhancing clinical outcomes in AML therapy and potentially enhancing the efficacy of anthracycline-based treatment regimens.

## 5. Conclusions

Numerous human cancers have been shown to produce GHRH, which acts as a growth factor through an autocrine/paracrine mechanism. In previous studies, we have shown that the GHRH antagonist MIA-602 suppresses the growth of several distinct human cancer cell lines in both in vivo and in vitro models. Additionally, our previous studies have demonstrated the role of MIA-602 in the inhibition of proliferation via the upregulation of proapoptotic genes. Our current study focuses on the role of MIA-602 in addressing resistance to both ATRA and ATO in the treatment of APL. Using the NB4 model cell line, we developed an in vitro model of resistance and found that these cells remain susceptible to the GHRH antagonist MIA-602, which exerts a distinct mechanism from that of ATRA and ATO. Furthermore, our study demonstrates the synergistic effects of MIA-602 when combined with anthracycline-based regimens in the treatment of AML utilizing the K-562 AML cell line. Our findings present a potential new therapeutic approach and augmentation strategy for the treatment of AML and APL.

## 6. Patents

Andrew Schally is listed as an inventor on patents for GHRH antagonists assigned to the University of Miami and VA.

## Data Availability

The data can be shared up on request.

## Supplementary Materials

The following supporting information can be downloaded at: https://www.mdpi.com/article/10.3390/cancers15123104/s1, File S1: Original western blots.
