# Supplementary figures and images for: The Application of GHRH Antagonist as a Treatment for Resistant APL
Source: Cancers (Basel). 2023 Jun 8;15(12):3104. doi: 10.3390/cancers15123104 (PMC10296252; doi:10.3390/cancers15123104)

## Slide 1
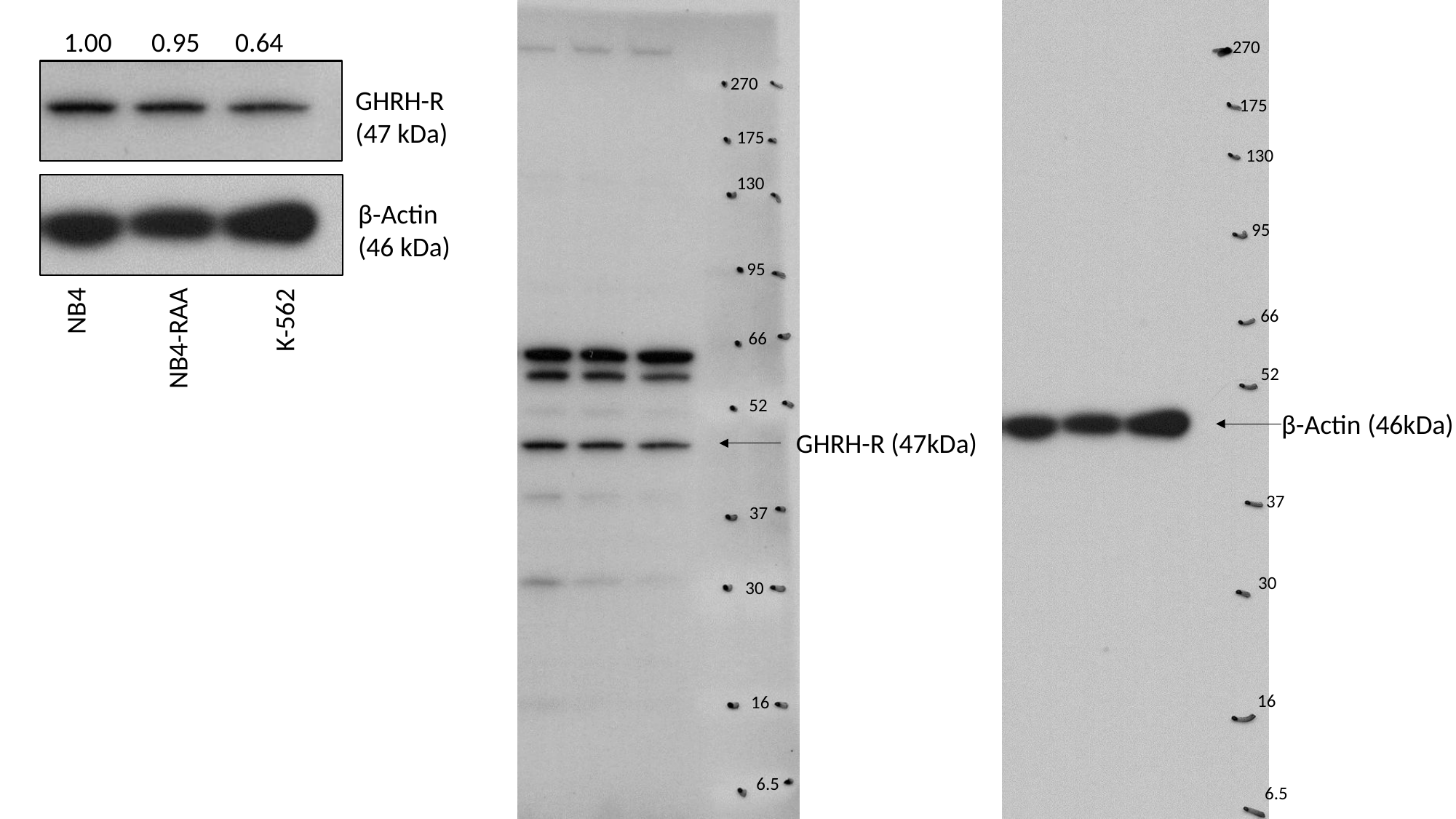

1.00
0.95
0.64
270
270
GHRH-R
(47 kDa)
175
175
130
130
β-Actin
(46 kDa)
95
95
NB4
K-562
66
NB4-RAA
66
52
52
β-Actin (46kDa)
GHRH-R (47kDa)
37
37
30
30
16
16
6.5
6.5

Supplement: Supplementary file 1 [file cancers-15-03104-s001.zip › cancers-2306410-File S1.pptx]
